# Promiscuous bacteria have staying power

**DOI:** 10.7554/eLife.30734

**Published:** 2017-09-08

**Authors:** Ruth C Massey, Daniel J Wilson

**Affiliations:** 1 School of Cellular and Molecular MedicineUniversity of BristolBristolUnited Kingdom; 2 Nuffield Department of MedicineUniversity of OxfordOxfordUnited Kingdom

**Keywords:** S. pneumoniae, GWAS, carriage duration, epidemiology, heritability, Other

## Abstract

Being able to take up DNA from their environment might allow pneumococcal bacteria to colonize the human nose and throat for longer periods of time.

**Related research article** Lees JA, Croucher NJ, Goldblatt D, Nosten F, Parkhill J, Turner C, Turner P, Bentley SD. 2017. Genome-wide identification of lineage and locus specific variation associated with pneumococcal carriage duration. *eLife* **6**:e26255. doi: 10.7554/eLife.26255

*Streptococcus pneumoniae* is a notorious bacterial pathogen hiding in plain sight. A common resident of the nose and throat, between 68% and 84% of young infants will carry this species at any given time (Turner et al., 2012). In most cases it causes no harm, yet the presence of pneumococci – as the bacteria are known – can predispose a person to life-threatening infections like pneumonia or meningitis. Indeed, pneumococci are responsible for around 10% of all deaths in young children around the world (O'Brien et al., 2009), with the vast majority of cases being in developing countries.

Research into *S. pneumoniae* is complicated because the species is a patchwork of distinctive strains and some of these strains remain in the nose and throat for longer than others. Now, in eLife, John Lees and Stephen Bentley – both at the Wellcome Trust Sanger Institute – and colleagues report that strains rendered impotent by a virus do not linger for as long as other strains (Lees et al., 2017).

Different pneumococci can be placed into groups called serotypes, based on the properties of their protective outer coating. Pneumococcal vaccines target the most harmful serotypes and vaccination reduces both the frequency of disease and symptomless colonization (Scott et al., 2012). People who are colonized often, or are colonized for long periods, have a greater risk of life-threatening infection (Simell et al., 2012). While the different serotypes were known to, on average, colonize people for different lengths of time (e.g. Abdullahi et al., 2012; Turner et al., 2012), it was not well understood how other factors such as the genetic background of the strain or its individual genes contributed to this variation in the duration of colonization.

Lees et al. focused on a high-risk population of infants from Maela, a camp near the Thailand-Myanmar border that provides refuge to more than 45,000 displaced people. Around one in four children in the camp developed bacterial pneumonia each year, with very severe pneumonia in around 8% of cases (Turner et al., 2013).

Using a combination of whole genome sequencing and sophisticated statistics, Lees et al. analyzed over 14,000 nose swab samples collected from almost 600 children in the camp over a two-year period (Figure 1). First, they removed noise from the epidemiological data to allow for the fact that the limited sensitivity of tests can make it appear that a continued episode of colonization is instead a series of shorter episodes. Second, they used statistics to determine how much a number of different variables – ranging from the age and infection history of the child to the serotype and genetic background of the bacteria – could explain the observed variability in the length of time the children remained colonized. Finally, they searched the genomic data to look for any links between individual genetic elements in the bacteria and the duration of colonization.

**Figure 1.**
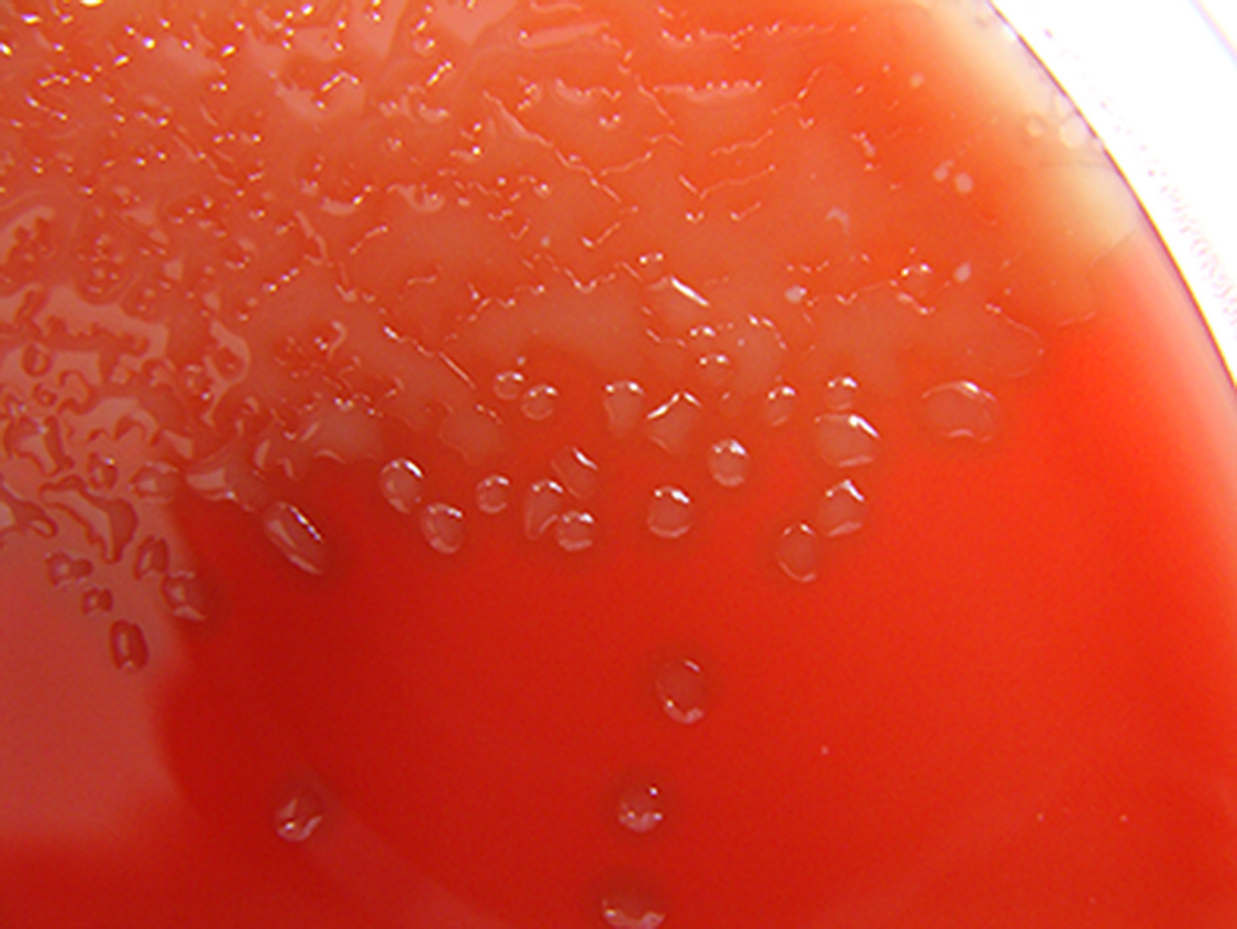
*Streptococcus pneumoniae* isolated from a child can be grown on blood agar. Pneumococci grow as glistening colonies on agar plates. Blood is included in the agar because it provides an enzyme that can neutralize the harmful amount of hydrogen peroxide that the bacteria naturally produce.

IMAGE CREDIT: Nathan Reading.

The most striking finding reported by Lees et al., however, owes more to lateral thinking than state-of-the-art statistics. Faced with a difficult-to-interpret association between duration and a rare version of a housekeeping gene in the bacteria, they reassessed their data. The housekeeping gene in question is found in some phages (viruses that infect bacteria), so the researchers asked whether phage integration in general was associated with the duration of colonization. Previous work had shown that phages regularly integrated into a particular pair of genes in pneumococci, referred to as *comYC* (Croucher et al., 2016). Pneumococci need the *comYC* genes to be able to take up DNA from their environment and integrate it into their genome, which can be thought of as a form of ‘bacterial sex’. Lees et al. saw that intact *comYC* genes were strongly associated with longer periods of carriage compared to *comYC* genes that had been disrupted by phages. This indicates that promiscuous pneumococci enjoy prolonged colonization of the nose and throat.

It is interesting to speculate why bacterial promiscuity might be wrapped up in the duration of colonization, which is perhaps linked to the extreme stresses pneumococci are exposed to in the human nose and throat. Not only do they have to survive the onslaught of the human immune system, but they also face a constant battle with the other microbes that also reside in this area of the human body. Surviving in this niche would likely require the pneumococci to evolve quickly to adapt to changing circumstances. As bacteria that could not take up DNA from their environment would be less able to evolve, this may explain the association between phage integration into *comYC* and the duration of colonization. Others have also argued that bacterial promiscuity protects directly against phage infection itself (Croucher et al., 2016). But whatever the selective forces resulting in the association between the phage and prolonged duration of colonization, this work provides compelling evidence in support of the benefits of bacterial promiscuity.

Lees et al. – who are based at the Sanger Institute, Imperial College London, University College London, Oxford University and Mahidol University – also provide a timely example of the potential of genome wide association studies to uncover new links between genotypes and phenotypes in bacteria. This is particularly heartening in view of the challenges posed by clonal reproduction in bacteria, which normally makes it difficult to tease apart the effects of individual genes in this kind of observational study (Earle et al., 2016). With the arrival of studies into important traits related to human health and disease that are not readily studied in the laboratory (e.g. Recker et al., 2017), we are now entering a new era of discovery into the genomic basis of diverse bacterial traits.

## References

[bib1] Abdullahi O, Karani A, Tigoi CC, Mugo D, Kungu S, Wanjiru E, Jomo J, Musyimi R, Lipsitch M, Scott JA (2012). Rates of acquisition and clearance of pneumococcal serotypes in the nasopharynges of children in Kilifi District, Kenya. The Journal of Infectious Diseases.

[bib2] Croucher NJ, Mostowy R, Wymant C, Turner P, Bentley SD, Fraser C (2016). Horizontal DNA transfer mechanisms of bacteria as weapons of intragenomic conflict. PLOS Biology.

[bib3] Earle SG, Wu CH, Charlesworth J, Stoesser N, Gordon NC, Walker TM, Spencer CCA, Iqbal Z, Clifton DA, Hopkins KL, Woodford N, Smith EG, Ismail N, Llewelyn MJ, Peto TE, Crook DW, McVean G, Walker AS, Wilson DJ (2016). Identifying lineage effects when controlling for population structure improves power in bacterial association studies. Nature Microbiology.

[bib4] Lees JA, Croucher NJ, Goldblatt D, Nosten F, Parkhill J, Turner C, Turner P, Bentley SD (2017). Genome-wide identification of lineage and locus specific variation associated with pneumococcal carriage duration. eLife.

[bib5] O'Brien KL, Wolfson LJ, Watt JP, Henkle E, Deloria-Knoll M, McCall N, Lee E, Mulholland K, Levine OS, Cherian T, Hib and Pneumococcal Global Burden of Disease Study Team (2009). Burden of disease caused by *Streptococcus pneumoniae* in children younger than 5 years: global estimates. The Lancet.

[bib6] Recker M, Laabei M, Toleman MS, Reuter S, Saunderson RB, Blane B, Török ME, Ouadi K, Stevens E, Yokoyama M, Steventon J, Thompson L, Milne G, Bayliss S, Bacon L, Peacock SJ, Massey RC (2017). Clonal differences in *Staphylococcus aureus* bacteraemia-associated mortality. Nature Microbiology.

[bib7] Scott JR, Millar EV, Lipsitch M, Moulton LH, Weatherholtz R, Perilla MJ, Jackson DM, Beall B, Craig MJ, Reid R, Santosham M, O'Brien KL (2012). Impact of more than a decade of pneumococcal conjugate vaccine use on carriage and invasive potential in Native American communities. The Journal of Infectious Diseases.

[bib8] Simell B, Auranen K, Käyhty H, Goldblatt D, Dagan R, O'Brien KL, Group PC, Pneumococcal Carriage Group (2012). The fundamental link between pneumococcal carriage and disease. Expert Review of Vaccines.

[bib9] Turner P, Turner C, Jankhot A, Helen N, Lee SJ, Day NP, White NJ, Nosten F, Goldblatt D (2012). A longitudinal study of *Streptococcus pneumoniae* carriage in a cohort of infants and their mothers on the Thailand-Myanmar border. PLoS ONE.

[bib10] Turner C, Turner P, Carrara V, Burgoine K, Tha Ler Htoo S, Watthanaworawit W, Day NP, White NJ, Goldblatt D, Nosten F (2013). High rates of pneumonia in children under two years of age in a South East Asian refugee population. PLoS ONE.

